# Acupuncture for opioid-induced constipation: Protocol for a systematic review and meta-analysis

**DOI:** 10.1097/MD.0000000000023352

**Published:** 2020-12-04

**Authors:** Pu Yang, Yuanchun Wang, Yingchun Xiao, Qiaolin Ma, Runhong Ma, Jing Mi, Jianrong Hui

**Affiliations:** a School of Acupuncture-Moxibustion and Tuina, Shaanxi University of Chinese Medicine, Xian; b The Affiliated Hospital of Shaanxi University of Chinese Medicine, Xianyang, Shaanxi Province, China.

**Keywords:** acupuncture, opioid-induced constipation, meta-analysis, systematic review, protocol

## Abstract

**Background:**

Opioid-induced constipation (OIC) is one of the most common complications of analgesic therapy for cancer pain patients who suffer moderate to severe pain. Acupuncture as an effective treatment in constipation has been widely applied. But its efficacy has not been assessed systematically. Thus, the purpose of this study is to provide a protocol to explore the efficacy and safety of acupuncture for OIC.

**Methods:**

Randomized Controlled Trials (RCTs) of acupuncture treatment for OIC in 4 Chinese electronic databases (China National Knowledge Infrastructure, Chinese Biological and Medical Database, China Scientific Journal Database, Wan-Fang Data) and 3 English electronic databases (PubMed, Embase, Cochrane Library) will be searched from their inception to September 31, 2020. RevMan 5.3 software and Stata 14.0 software will be used for meta-analysis, EndNote X9.2 and Cochrane Risk of Bias Tool will be used for literature screening and quality assessment.

**Results:**

This study will present an assessment of the efficacy and safety of acupuncture treatment for OIC patients through summarize high-quality clinical evidence.

**Conclusion:**

The conclusion of our systematic review and meta-analysis may provide evidence of whether acupuncture treatment is beneficial to patients with OIC.

INPLASY registration number: INPLASY2020100026.

## 1 Introduction

Opioids are powerful analgesics used for the treatment of acute and chronic pain.^[1]^ WHO also proposed a three-step analgesic ladder for alleviating moderate-to-severe cancer pain by used opioids.^[2]^ Though the WHO three-step ladder has been recognized and used widely around the world for many years, Side-effects are widespread, and among the most troublesome are those linked to opioids-induced bowel dysfunction, which particularly includes opioids-induced constipation (OIC).^[3,4]^ OIC has a negative influence on work productivity, quality of life, and increased national health expenditures.^[5]^ The Rome IV standard defines OIC as new or upgraded symptoms of constipation that appear at the beginning, change, or increase of opioid therapy, and have further clinical features, such as a feeling of incomplete emptying and less than 3 spontaneous bowel movements per week.^[6]^

OIC is the most common and bothersome problem for patients with chronic taking opioids therapy, it affects 60% to 90% of cancer patients with opioids.^[7,8]^ It has been reported that about 215 million prescriptions for opioids in the United States in 2019.^[9]^ OIC occurs primarily related to μ-opioid receptor activation in the gut that reduces rectal sensation, decreases peristalsis and increases colonic fluid absorption. This results in harder stools.^[10]^ The National Comprehensive Cancer Network (NCCN) guidelines referred that the prevention and treatment of adverse reactions are an important part of the analgesic therapy plan. Once opioids are used, the prescription laxatives should be implemented to treatment OIC.^[11]^ However, laxatives do not target the underlying cause of opioid binding to the μ-receptors in the enteric system and as such are not very effective at managing OIC.^[12,13]^ Accordingly, it is essential to find an alternative treatment.

Acupuncture is highly valued in traditional Chinese medicine and has a long historical source of more than 2500 years, and maybe a utility non-drug therapy option for OIC.^[14–16]^ However, acupuncture as an adjunctive therapy exists a doubt in mainstream oncology. Therefore, it is necessary to evaluate the efficacy and safety of acupuncture in treating OIC through systematic review and meta-analysis, which intend to offer a reliable basis for clinical practice.

## 2 Methods

### 2.1 Design and registration of the review

This study has been registered on INPLASY and the registration number is INPLASY2020100026 and the protocol follows the Cochrane Handbook for Systematic Reviews and the Preferred Reporting Items for Systematic Reviews and Meta-Analysis Protocols (PRISMA-P) statement guidelines.

### 2.2 Inclusion criteria

#### 2.2.1 Type of studies

All randomized controlled trials (RCTs) of acupuncture therapy for OIC will be included in the study, while animal experiments, cluster RCTs, reviews, and case reports will be excluded.

#### 2.2.2 Types of participants

The study will include patients who were clinically diagnosed with OIC. There is no restriction on age, gender, or nationality. Besides, the diagnostic criteria are based on the Rome III criteria.^[17]^

#### 2.2.3 Types of intervention

The patients in the intervention group adopt acupuncture and related treatments, regardless of needle material, acupoint selection, duration of treatment, acupuncture manipulation, while the patients in the control group are treated with drugs, placebo, sham acupuncture, or other conventional therapy, either.

### 2.3 Types of outcome measures

#### 2.3.1 Primary outcomes

The primary efficacy outcomes measure will be as follows: changes in the Bowel Function Index (BFI) score or Cleveland Constipation Score (CCS).

#### 2.3.2 Secondary outcomes

The secondary outcome measures will include the Patient Assessment of Constipation Quality of Life (PAC-QOL) questionnaire, adverse effects linked to interventions.

### 2.4 Exclusion criteria

The following conditions in the literature will be excluded: repeated literature; incomplete data; inappropriate design method.

### 2.5 Search strategy

We will search for PubMed, Embase, Cochrane Library, CNKI, WF, VIP, CBM literature databases from its inception to September 2020 with a language restriction on Chinese or English. The details of the search strategy for PubMed are shown in Table 1.

**Table 1:** Search strategy for the PubMed database.

Number	Search terms
#1	opioid-induced constipation.
#2	opiate-induced constipation.
#3	narcotic bowel syndrome.
#4	opioid-induced bowel dysfunction.
#5	OIC
#6	NBS
#7	OIBD
#8	#1 OR #2 OR #3 OR #4 OR #5 OR #6 OR #7
#9	acupuncture therapy
#10	acupuncture treatment
#11	pharmacoacupuncture therapy
#12	acupotomy
#13	electroacupuncture
#14	acupuncture-moxibustion
#15	auricular acupuncture
#16	embedded thread therapy
#17	moxibustion
#18	catgut embedding
#19	warm needling
#20	#9 OR #10 OR #11 OR #12 OR #13 OR #14 OR #15 OR #16 OR #17 OR #18 OR #19
#21	randomized controlled trial
#22	controlled clinical trial
#23	randomized
#24	clinical trial
#25	#21 OR #22 OR #23 OR #24
#26	Exp animals/ Not humans
#27	#25 Not #26
#28	#8 And #20 And #27

### 2.6 Data collection and analysis

#### 2.6.1 Study selection

**Figure 1:**
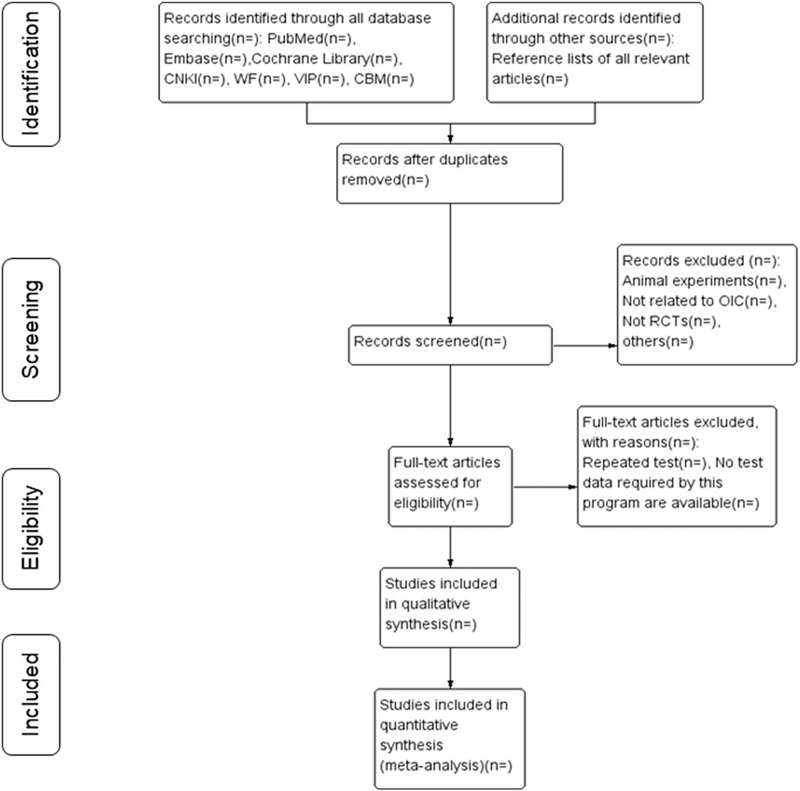
Flow chart of the search process.

Two of the researchers (PY and YCX) will be extract data independently by reading all titles and abstracts. The screen results are inconsistent will be settled through discussion between the above 2 authors. If their discussion still cannot reach accordance, another author (JRH) will make a final decision of eligible study selection. We will adopt EndNote X9.2 software to conduct a preliminary elimination of duplicate literature, then according to the inclusion and exclusion criteria, a brief screening will perform by reading the titles, abstracts, and keywords of the literature. Besides, we will review the full text to determine the final eligible literature based on details in the articles. The selection procedure of studies is summarized in the following PRISMA flow diagram (Fig. 1).

#### 2.6.2 Data extraction

All information will be extracted by 2 of the independent authors (QLM and RHM) according to predetermined criteria form. Disagreement will be resolved by consulting a third author (YCW), and the extracted data as following: first author, publication date, country, sample size, gender, mean age, details of interventions, treatment courses, follow-up, outcomes, and adverse event. If the information on the papers is unclear, we will contact the author by sending an email.

#### 2.6.3 Risk of bias assessment

The risk of bias assessment of the included RCTs will be evaluated by using the risk of bias assessment tool of the Cochrane Handbook, version 5.1.0, which includes 7 items as following: random sequence generation, allocation concealment, blinding of participants and personnel, blinding of outcome assessment, incomplete outcome data, selective reporting, other bias. This evaluation will be conducted by 2 independent reviewers (JM and PY) according to a judgment for literature that will be categorized as low bias, unclear bias, or high bias.

#### 2.6.4 Data synthesis and analysis

RevMan 5.3 software will be used for data synthesis and analysis. When the outcome data is a binary variable, select the relative risk (RR) as the effect scale; when the outcome data is a continuous variable, use the mean difference (MD) and standardized mean difference (SMD) as an effect scale, both calculated by 95% confidence interval (CI).

#### 2.6.5 Assessment of heterogeneity

The heterogeneity test adopts the χ^2^ test, and *I*^2^ statistic will be used to evaluate heterogeneity. If *P* > .1 and *I*^2^ < 50%, the fixed effects model will be used. If *P* ≤ .1 and *I*^2^ ≥ 50%, the random-effects model will be used.

#### 2.6.6 Analysis of subgroups

If significant heterogeneity is detected between a group of studies, subgroup analysis will be performed based on acupuncture types, countries, treatment courses, the control group intervention measures.

#### 2.6.7 Sensitivity analysis

If the heterogeneity is significant, we will conduct a sensitivity analysis according to eliminating each of the included studies one by one, and changing the effect scale of studies to evaluate the robustness and quality of the conclusion in the studies.

#### 2.6.8 Assessment of reporting biases

First, if there are more than 10 studies are included, we will draw a funnel plot to analyze publication bias via RevMan 5.3 software, after Egger test and Begg test will be carried out to explore the potential publication bias of studies by using Stata 14.0 software if the funnel plot is asymmetric.

#### 2.6.9 Ethics and dissemination

This meta-analysis and systematic review protocol will not involve ethical approval because where there not contain individual patient data. We will publish this study in peer-reviewed journals and conference presentations, which provide evidence of the efficacy and safety of acupuncture treatment for OIC.

## 3 Discussion

Constipation is the most common and long-term intolerant adverse reaction of opioids, it seriously affects patient quality of life and cannot currently be effectively treated.^[18]^ OIC is caused by the action exerted on opioid receptors in the gastrointestinal tract, in which the mechanism differs from idiopathic constipation. A study has shown that lifestyle changes and over-the-counter drugs are first-line treatments. ^[19]^ But in fact, there is still no satisfactory effect and effective alternative therapy in some cases. In recent years, studies of animal experiments show that acupuncture can improve gastrointestinal motility and expression of 5-HT by adjusting nerve stimulation.^[20–22]^ However, the efficacy has not been recognized by the clinical guidance and medical organizations, meanwhile, there is not a systematic review about acupuncture for OIC to investigate the clinical efficacy and safety. So we conduct this study to provide a basis of evidence-based medicine and help clinicians make decisions in practice.

As far as we know, it is the first time to conduct a systematic review and meta-analysis of acupuncture treatment for OIC, it demonstrates acupuncture has better results in clinical outcomes than non-acupuncture therapy. On the other hand, this study has some limitations, involving the quality of included literature, the inconsistency of acupuncture types, the methodology of studies, language limitation, which may lead to the high heterogeneity.

## Author contributions

**Conceptualization:** Pu Yang.

**Methodology:** Pu Yang, Yuanchun Wang.

**Formal analysis:** Yingchun Xiao, Qiaolin Ma, Runhong Ma, Jing Mi.

**Supervision:** Jianrong Hui.

**Writing – original draft:** Pu Yang, Yuanchun Wang.

**Writing – review & editing:** Pu Yang, Yuanchun Wang, Jianrong Hui.

## References

[1] Farmer AD Holt CB Downes TJ Pathophysiology, diagnosis, and management of opioid-induced constipation. Lancet Gastroenterol Hepatol 2018;3:203–12. 29870734 10.1016/S2468-1253(18)30008-6

[2] Wiffen PJ Derry S Moore RA Oral paracetamol (acetaminophen) for cancer pain. Cochrane Database Syst Rev 2017;7:Cd012637. 28700092 10.1002/14651858.CD012637.pub2 PMC6369932

[3] Bandieri E Romero M Ripamonti CI Randomized trial of low-dose morphine versus weak opioids in moderate cancer pain. J Clin Oncol 2016;34:436–42. 26644526 10.1200/JCO.2015.61.0733

[4] Müller-Lissner S Bassotti G Coffin B Opioid-induced constipation and bowel dysfunction: a clinical guideline. Pain Med 2017;18:1837–63. 28034973 10.1093/pm/pnw255 PMC5914368

[5] Farmer AD Drewes AM Chiarioni G Pathophysiology and management of opioid-induced constipation: European expert consensus statement. United European Gastroenterol J 2019;7:7–20. 10.1177/2050640618818305 PMC6374852 30788113

[6] Crockett SD Greer KB Heidelbaugh JJ American Gastroenterological Association institute guideline on the medical management of opioid-induced constipation. Gastroenterology 2019;156:218–26. 30340754 10.1053/j.gastro.2018.07.016

[7] Larkin PJ Cherny NI La Carpia D Diagnosis, assessment and management of constipation in advanced cancer: ESMO Clinical Practice Guidelines. Ann Oncol 2018;29:iv111–25. 10.1093/annonc/mdy148 30016389

[8] Mesía R Virizuela Echaburu JA Gómez J Opioid-induced constipation in oncological patients: new strategies of management. Curr Treat Options Oncol 2019;20:91. 31853656 10.1007/s11864-019-0686-6 PMC6920224

[9] Crockett S Greer KB Sultan S Opioid-Induced Constipation (OIC) guideline. Gastroenterology 2019;156:228. 30389393 10.1053/j.gastro.2018.10.044

[10] Rao VL Micic D Davis AM Medical management of opioid-induced constipation. JAMA 2019;322:2241–2. 10.1001/jama.2019.15852 31682706

[11] Prichard D Norton C Bharucha AE Management of opioid-induced constipation. Br J Nurs 2016;25: S4-5, s8-11. 10.12968/bjon.2016.25.10.S4 27231750

[12] Sera L McPherson ML Management of opioid-induced constipation in hospice patients. Am J Hosp Palliat Care 2018;35:330–5. 28423917 10.1177/1049909117705379

[13] Kumar L Barker C Emmanuel A Opioid-induced constipation: pathophysiology, clinical consequences, and management. Gastroenterol Res Pract 2014;2014:141737. 24883055 10.1155/2014/141737 PMC4027019

[14] Lu W Rosenthal DS Acupuncture for cancer pain and related symptoms. Curr Pain Headache Rep 2013;17:321. 23338773 10.1007/s11916-013-0321-3 PMC4008096

[15] Zhang FL Lin HS He QY Effect of electro-acupuncture in treating morphine sulfate caused constipation in tumor patients. Zhongguo Zhong Xi Yi Jie He Za Zhi 2009;29:922–5. 20073226

[16] Zhu HD Gong Z Hu BW The Efficacy and safety of transcutaneous acupoint interferential current stimulation for cancer pain patients with opioid-induced constipation: a prospective randomized controlled study. Integr Cancer Ther 2018;17:437–43. 29076387 10.1177/1534735417734910 PMC6041914

[17] Gaertner J Siemens W Camilleri M Definitions and outcome measures of clinical trials regarding opioid-induced constipation: a systematic review. J Clin Gastroenterol 2015;49:9–16. 25356996 10.1097/MCG.0000000000000246

[18] Cai H Zhou Q Bao G Transcutaneous electrical nerve stimulation of acupuncture points enhances therapeutic effects of oral lactulose solution on opioid-induced constipation. J Int Med Res 2019;47:6337–48. 31774002 10.1177/0300060519874539 PMC7045659

[19] Webster LR Opioid-induced constipation. Pain Med 2015;16: Suppl 1: S16–21. 26461071 10.1111/pme.12911

[20] Hutson JM Dughetti L Stathopoulos L Transabdominal electrical stimulation (TES) for the treatment of slow-transit constipation (STC). Pediatr Surg Int 2015;31:445–51. 25672282 10.1007/s00383-015-3681-4

[21] Wang X Yang B Yin J Electroacupuncture via chronically implanted electrodes improves gastrointestinal motility by balancing sympathovagal activities in a rat model of constipation. Am J Physiol Gastrointest Liver Physiol 2019;316:G797–805. 30920306 10.1152/ajpgi.00018.2018

[22] Zhu X Liu Z Qu H The effect and mechanism of electroacupuncture at LI11 and ST37 on constipation in a rat model. Acupunct Med 2016;34:194–200. 26561562 10.1136/acupmed-2015-010897 PMC4941155

